# *Prmt5* is required for germ cell survival during spermatogenesis in mice

**DOI:** 10.1038/srep11031

**Published:** 2015-06-15

**Authors:** Yanbo Wang, Tianxiang Zhu, Qiuling Li, Chunyi Liu, Feng Han, Min Chen, Lianjun Zhang, Xiuhong Cui, Yan Qin, Shilai Bao, Fei Gao

**Affiliations:** 1 State Key Laboratory of Reproductive Biology, Institute of Zoology, Chinese Academy of Sciences, Beijing 100101, China; 2 University of Chinese Academy of Sciences, Beijing, China; 3 State Key Laboratory of Molecular and Developmental Biology, Institute of Genetics and Developmental Biology, Chinese Academy of Sciences, Beijing 100101, China; 4 School of Medicine, Zhejiang University, Hangzhou 310058, China

## Abstract

During germ cell development, epigenetic modifications undergo extensive remodeling. Abnormal epigenetic modifications usually result in germ cell loss and reproductive defect. *Prmt5* (Protein arginine methyltransferase 5) encodes a protein arginine methyltransferase which has been demonstrated to play important roles in germ cell development during embryonic stages. In the present study, we found that *Prmt5* was also abundantly expressed in male germ cells after birth. Inactivation of this gene by crossing with *Stra8-Cre* transgenic mice resulted in germ cell loss during spermatogenesis. Further study revealed that the germ cell development was grossly normal before P10. However, most of the germ cells in *Prmt5*^*Δ/f*^*; Stra8-Cre* mice were blocked at meiotic stage. The expression of meiosis associated genes was reduced in *Prmt5*^*Δ/f*^*; Stra8-Cre* testes compared to control testes at P10. γH2AX was detected in sex body of control germ cells at P12, whereas multiple foci were observed in *Prmt5*-deficient germ cells. Further study revealed that H4R3me2s was virtually absent in germ cells after *Prmt5* inactivation. The results of this study indicate that *Prmt5* also plays important roles in germ cell development during spermatogenesis.

Epigenetic modification is one of the important mechanisms regulating the gene expression, which is involved in a number of biological processes. Epigenetic processes include DNA methylation, histone modifications, and chromatin remodeling^1^. In mammals, germ cell is a special cell type which is different from other cell types that constitute the animal body. During germ cell development, both genetic and epigenetic mechanisms are involved^2^,^3^,^4^. In mice, primordial germ cells (PGCs) first emerge inside the extra-embryonic mesoderm at around E7.25^5^,^6^. The somatic gene expression program needs to be suppressed in the PGC precursors, and epigenetic modifications might be important for this process. After arriving at the genital ridge by E11.5, PGCs will undergo extensive epigenetic reprogramming. The parental imprints are erased and the gender-specific new imprints are re-established at later developmental stages^7^. Epigenetic modifications also play important roles in later stage of germ cell development, including meiosis initiation and maturation of gametes. In male germ cells, proper regulation of epigenetic processes not only ensure proper sperm function, but also important for proper embryonic development. It has been demonstrated that aberrant epigenetic modification in spermatogenesis has a profound effect on both male fertility and embryonic development^8^.

Post-translational histone modifications include methylation, acetylation, phosphorylation, ubiquitylation and sumoylation. Methylation is one of the most prevalent histone modifications monitored by histone methyltransferases^9^. Arginine methylation is catalyzed by protein arginine methyltransferases (PRMTs)^10^. PRMT family members play pivotal roles in the regulation of diverse cellular processes ranging from transcription and RNA processing to signaling transduction, cell differentiation, apoptosis and tumorigenesis^11^,^12^. *Prmt5* belongs to the PRMT family and is responsible for the formation of symmetric dimethylarginine (SMDA) in arginine-rich protein motifs^13^. It has been reported that *Prmt5* is essential for maintaining the pluripotency of mouse embryonic stem cells (ES). Deletion of *Prmt5* results in the down-regulation of pluripotency transcription factors and causes embryonic lethality before implantation^14^. However, *Prmt5* is not required to maintain pluripotency in human ES cells^15^. *Prmt5* is also expressed in primordial germ cells (PGCs) and directs histone arginine methylation in mouse germ cells^16^, recent studies found that inactivation of *Prmt5* in PGCs using *Blimp1-Cre* resulted in germ cells death before E12.5^17^,^18^, suggesting that *Prmt5* plays essential roles in PGCs survival.

In this study, we found that *Prmt5* was also abundantly expressed in germ cells of adult testis, suggesting that histone methylation probably also plays roles in spermatogenesis. To investigate the functions of *Prmt5* in later stage of germ cell development, it was specifically inactivated in male germ cells by crossing *Prmt5*^*flox*^ mice with *Stra8-Cre* transgenic mice. We found that the germ cells were gradually lost after day 12 and very few germ cells were survived in adult testes. The results of this study indicate that *Prmt5* is required for male germ cell survival during spermatogenesis.

## Results

### *Prmt5* was expressed in the germ cells of testes during spermatogenesis

**Figure 1:**
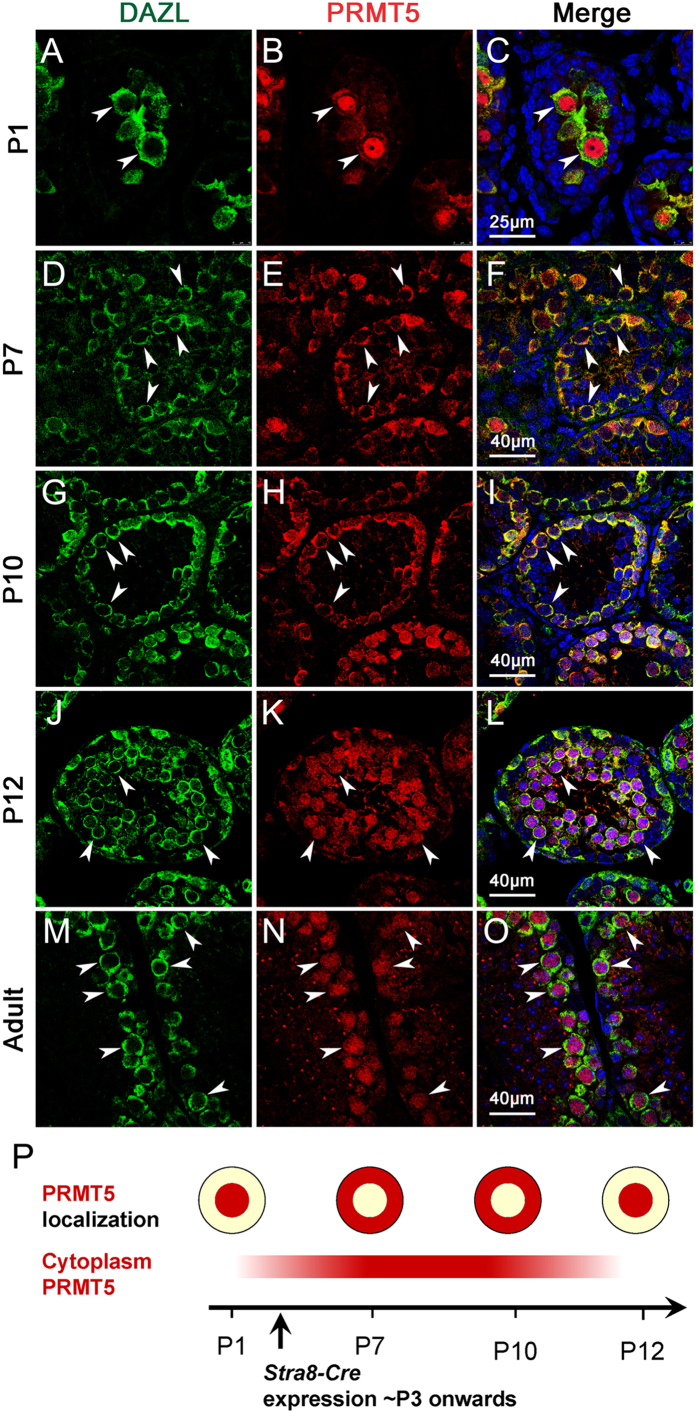
The expression of Prmt5 in postnatal male germ cells was dynamic. Immunofluorescence staining for PRMT5 (red) in testes at P1(**B**, **C**), P7(**E**, **F**), P10(**H**, **I**), P12(**K**, **L**) and adult(**N**, **O**). Germ cells were labeled with anti-DAZL antibody (**A**, **D**, **G**, **J**, **M**, green, white arrowheads). The nucleus were stained with DAPI (blue). **P**. Schematic diagram of nuclear-cytoplasm translocation of PRMT5 in male germ cells.

It has been reported that *Prmt5* is abundantly expressed in the germ cells during embryonic stages, and inactivation of *Prmt5* in PGCs results in germ cell loss in both male and female gonads^16^,^17^,^18^. In this study, the expression of *Prmt5* in testes after birth was examined by immunofluorescence. As shown in Fig. 1, *Prmt5* protein was localized in the nucleus of *Dazl*-posotive germ cells at P1 (B, C, white arrowheads). It was continually expressed in germ cells at P7 (E, F, white arrowheads) and P10 (H, I, white arrowheads), whereas the signal was mainly detected in cytoplasm at these stages. Interestingly, *Prmt5* protein was translocated from cytoplasm to the nucleus of germ cells at P12 (K, L, white arrowheads). In adult testes, *Prmt5* protein was abundantly expressed in the nucleus of spermatocytes (N, O, white arrowheads). The dynamics of nuclear-cytoplasm translocation of PRMT5 in germ cells postnatally was illustrated with the schematic diagram (Fig. 1P). These results indicate that *Prmt5* is continually expressed in male germ cells postnatally, and its location is dynamic along with germ cell development.

### Inactivation of *Prmt5* in germ cells resulted in germ cell loss and the defect of spermatogenesis

**Figure 2:**
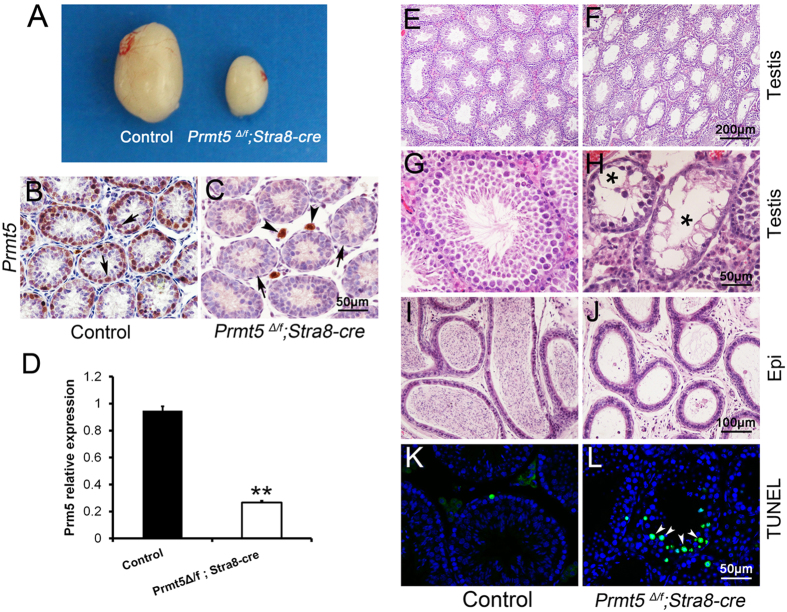
Deletion of Prmt5 in the testes by Stra8-Cre resulted in massive germ cell loss. The size of testes from adult *Prmt5*^*Δ/f*^; *Stra8-Cre* mice (**A**, right) was significantly smaller than that of control littermates (**A**, left). The protein (**C**) and mRNA (**D**) levels of *Prmt5* was dramatically reduced in *Prmt5*^*Δ/f*^; *Stra8-Cre* at P10. Most of seminiferous tubules were atrophic in adult *Prmt5*^*Δ/f*^; *Stra8-Cre* testes (**F, H**, asterisks). No mature sperm were observed in the epididymis of *Prmt5*-deficient males **(J**). The number of apoptotic cells was significantly increased in *Prmt5*^*Δ/f*^; *Stra8-Cre* testes (**L**, white arrowheads). **p < 0.01.

To examine the functions of *Prmt5* in germ cells at later developmental stages, *Prmt5*^*flox*^ mice were crossed with *Stra8-Cre* transgenic mice in which Cre is activated in germ cells of testes at approximately 3 days after birth^19^. *Prmt5*^*Δ/f*^; *Stra8-Cre* mice were grossly normal, no developmental defects were observed until 4 month of age. However, the size of testes from adult *Prmt5*^*Δ/f*^; *Stra8-Cre* males (Fig. 2A, right) was significantly smaller than that of control littermates (Fig. 2A , left) and most of seminiferous tubules were atrophic (Fig. 2F). High magnification images showed that very few germ cells were survived in the atrophic tubules (Fig. 2H, asterisks). In control mice, the caudal epididymis was filled with mature sperm (Fig. 2I), whereas no sperm was observed in epididymis from *Prmt5*^*Δ/f*^; *Stra8-Cre* males (Fig. 2J). A large number of TUNEL-positive apoptotic cells were noted in the seminiferous tubules of *Prmt5*^*Δ/f*^; *Stra8-Cre* mice (Fig. 2L, white arrowheads), but not in the control testes (Fig. 2K). These results indicate that *Prmt5* plays important roles in spermatogenesis, and inactivation of this gene results in germ cell death.

To examine the efficiency of *Stra8-Cre* mediated deletion of *Prmt5* gene, the expression of *Prmt5* was examined by real-time PCR and immunohistochemistry. As shown in Fig. 1, *Prmt5* was abundantly expressed in the germ cells of control testes at P10 (B, black arrows), whereas no PRMT5 was detected in the germ cells from *Prmt5*^*Δ/f*^; *Stra8-Cre* mice (C, black arrows) and only Leydig cells (C, black arrowheads) were labeled with anti-PRMT5 antibody. The results of immunofluorescence also showed that Prmt5 protein was absent in the germ cells of *Prmt5*^*Δ/f*^; *Stra8-Cre* testes at P7 (Fig. S1, E, F, white arrowheads). The mRNA level of *Prmt5* was reduced approximately 70% in *Prmt5*^*Δ/f*^; *Stra8-Cre* testes at P10 (Fig. 2D), indicating that *Prmt5* is deleted in germ cells with high efficiency.

### The germ cells were gradually lost in *Prmt5* ^*Δ/f*^ *; Stra8-Cre* testes from P12 onwards

**Figure 3:**
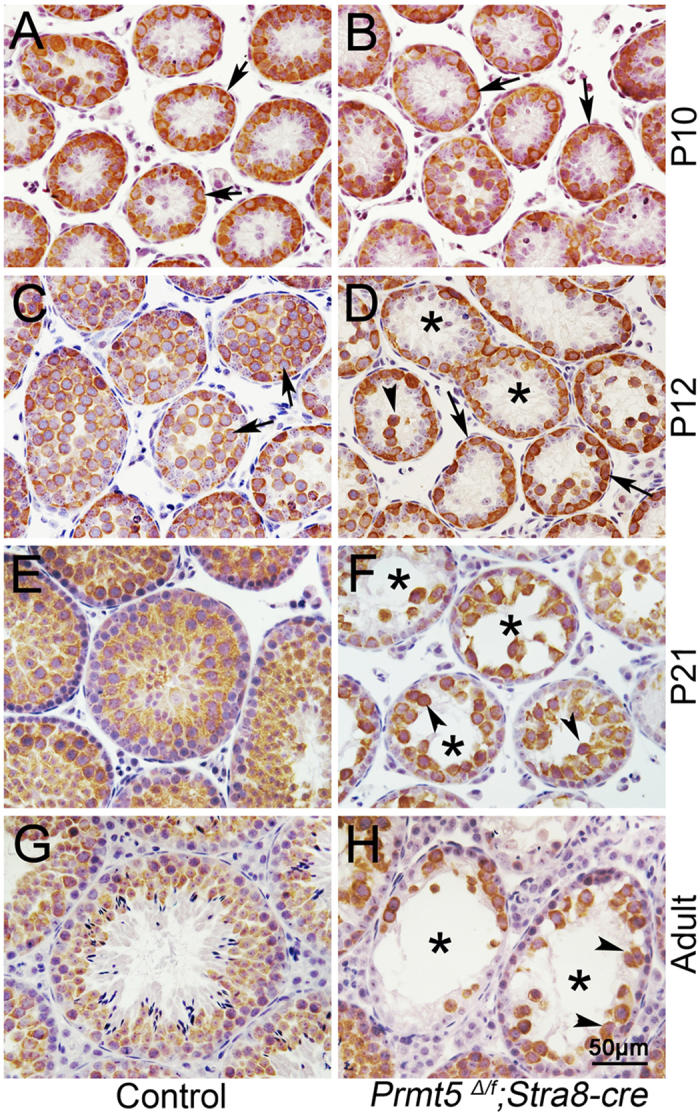
The germ cells were gradually lost in Prmt5Δ/f; Stra8-Cre testes from P12 onwards. Germ cells were labeled with antibody against MVH (brown, black arrow). *MVH*-positive germ cells were localized at the peripheral region of seminiferous tubules in both control (**A**, black arrows) and *Prmt5*^*Δ/f*^*; Stra8-Cre* mice (**B**, black arrows) at P10. The germ cells were observed in the lumen of seminiferous tubules from P12 to adult stage in control mice (**C**, **E**, **G**). By contrast, single layer of germ cells were observed at the peripheral region in most of seminiferous tubules (**D**, asterisks) of *Prmt5*^*Δ/f*^*; Stra8-Cre* testes at P12. The number of germ cells was dramatically reduced at P21 (F, black arrowheads) and only a few germ cells were observed in the seminiferous tubules in adult *Prmt5*^*Δ/f*^*; Stra8-Cre* testes (**H**, black arrowheads).

To further explore the underlying mechanisms which cause the defects of spermatogenesis in *Prmt5*^*Δ/f*^*; Stra8-Cre* mice, the histology of testes was examined by H&E staining and immunohistochemistry at early developmental stages. We found that the testes from *Prmt5*^*Δ/f*^; *Stra8-Cre* mice (Fig. S2B) was grossly normal at P10 compared to control testes (Fig. S2A). One layer of MVH-positive germ cells were observed at the peripheral region of seminiferous tubules in both control (Fig. 3A, black arrows) and *Prmt5*^*Δ/f*^*; Stra8-Cre* testes (Fig. 3B, black arrows) at this stage. Aberrant seminiferous tubules were first noted in *Prmt5*^*Δ/f*^; *Stra8-Cre* testes (Fig. S2D, asterisks) at P12, and atrophic tubules (asterisks) were observed in *Prmt5*-deficient testes at P14 (Fig. S2F, asterisks) and P21 (Fig. S2H, asterisks). Immunohistochemical results showed that the germ cells were localized in the lumen of seminiferous tubules from P12 to adult stage, and the number was gradually increased (Fig. 3C,E,G). By contrast, a single layer of germ cells were still located at the peripheral region in most of seminiferous tubules (Fig. 3D, asterisks) of *Prmt5*^*Δ/f*^*; Stra8-Cre* testes at P12. The number of germ cells was dramatically reduced at P21 (Fig. 3F, black arrowheads) and only a few germ cells were observed in the seminiferous tubules in testes of adult *Prmt5*^*Δ/f*^*; Stra8-Cre* males (Fig. 3H, black arrowheads). These results indicate that the germ cells were gradually lost from P12 after *Prmt5* inactivation which causes the defect of spermatogenesis in *Prmt5*^*Δ/f*^*; Stra8-Cre* mice.

### Aberrant meiosis was observed in *Prmt5-*deficient germ cells

**Figure 4:**
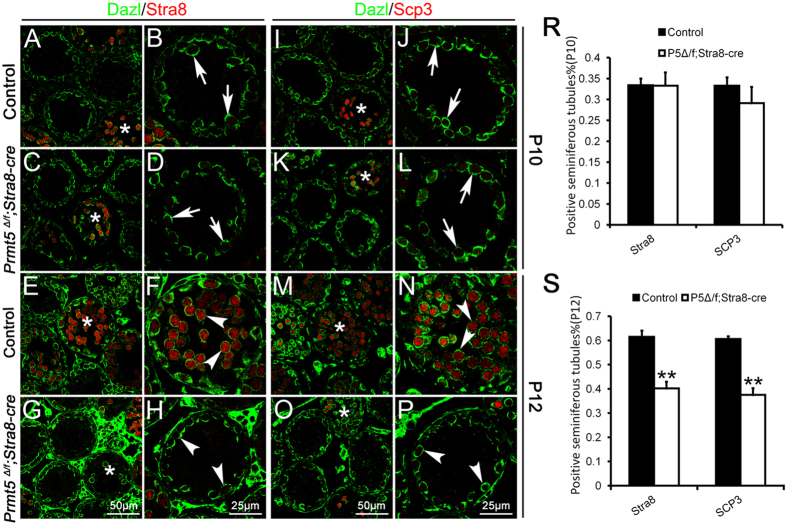
Aberrant germ cell meiosis was observed in Prmt5Δ/f; Stra8-Cre mice. The germ cells were labeled with Dazl (green) and meiotic germ cells were labeled with Stra8 (red) and Scp3 (red). In control testes, *Stra8*-positive (**A**, asterisks) and *Scp3*-positive (**I**, asterisks) germ cells were only detected in a small portion of seminiferous tubules at P10. *Stra8*-positive (C, asterisks) and *Scp3*-positive (**K**, asterisks) germ cells were also observed in seminiferous tubules of *Prmt5*^*Δ/f*^*; Stra8-Cre* mice at P10, and no statistical difference was noted compared to control mice (**R**). *Stra8*-positive (**E**, asterisks; **F**, white arrowheads) and *Scp3*-positive (**M**, asterisks; **N**, white arrowheads) germ cells were detected in most of seminiferous tubules of control testes at P12. The number of tubules with *Stra8*-positive (**G**) and *Scp3*-positive (**O**) germ cells was significantly reduced in *Prmt5*^*Δ/f*^*; Stra8-Cre* testes at P12 (**S**).

To examine whether the germ cell meiosis is affect after *Prmt5* inactivation, the expression of meiosis-associated genes was analyzed by immunofluorescence and real-time PCR analysis. *Stra8*-positive and *Scp3*-positive germ cells were detected in both control (Fig. 4A,I, asterisks) and *Prmt5*^*Δ/f*^*; Stra8-Cre* testes (Fig. 4C,K, asterisks) at P10, and the percentage of seminiferous tubules containing meiotic germ cells was not changed between control and *Prmt5*^*Δ/f*^*; Stra8-Cre* mice (Fig. 4R). *Stra8*-positive (Fig. 4E, asterisks; Fig. 4F, white arrowheads) and *Scp*3-positive (Fig. 4M, asterisks; Fig. 4N, white arrowheads) germ cells were detected in most of seminiferous tubules of control testes at P12. By contrast, the number of tubules with *Stra8*-positive (Fig. 4G, asterisks) and *Scp3*-positive (Fig. 4O, asterisks) germ cells was significantly reduced in *Prmt5*^*Δ/f*^*; Stra8-Cre* testes (Fig. 4S). The expression of Dmc1 and γH2AX was also examined by immunofluorescence. In control testes, Dmc1 protein (Fig. S3A, red) was detected in most of germ cells at P12, and co-localized with Scp3 (Fig. S3A, green). γH2AX (green) was mainly observed in sex body (Fig. S3D) of control germ cells. Dmc1 protein (Fig. S3 B, red) was also detected in a small number of germ cells in *Prmt5*-deficient germ cells, and co-localized with Scp3 (Fig. S3B, green). A small portion of germ cells in *Prmt5*^*Δ/f*^*; Stra8-Cre* testes were also γH2AX (green) positive, whereas multiple foci were noted (Fig. S3E). Dmc1 (Fig. S3C) and γH2AX (Fig. S3F) protein was absent in most of germ cells from *Prmt5*^*Δ/f*^*; Stra8-Cre* testes.

### The differentially expressed genes in *Prmt5* ^*Δ/f*^ *; Stra8-Cre* testes

**Figure 5:**
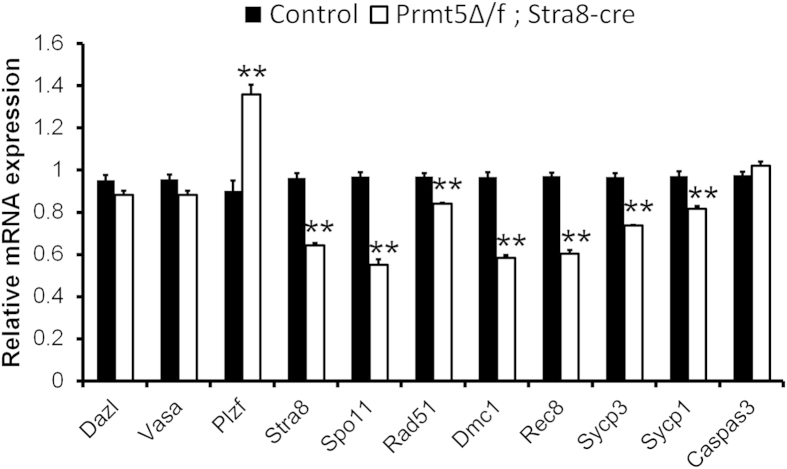
The expression of meiosis-associated genes was decreased in Prmt5Δ/f; Stra8-Cre testes at P10. The expression of germ cell specific and meiosis-associated genes was examined by real-time PCR analysis. The mRNA level of *Dazl* and *Vasa* was not changed between control and *Prmt5*-deficient testes, whereas the expression of meiosis-associated genes *Stra8*, *Spo11*, *Dmc1*, and *Rec8* was significantly reduced in testes of *Prmt5*^*Δ/f*^*; Stra8-Cre* testes compared to control testes. **p < 0.01.

The differentially expressed genes between control and *Prmt5*^*Δ/f*^*; Stra8-Cre* testes at P10 was examined by real-time PCR analysis. As shown in Fig. 5, the mRNA levels of meiosis associated genes (e.g. *Stra8, Spo11, Rad51, Scp1, Scp3, Dmc1, Rec8*) were significantly reduced in testes from *Prmt5*^*Δ/f*^*; Stra8-Cre* males. By contrast, the expression of germ cell specific genes, *Dazl* and *Vasa*, were not decreased in *Prmt5*-deficient germ cells. Interestingly, the mRNA level of *Plzf* was significantly increased in *Prmt5*^*Δ/f*^*; Stra8-Cre* testes. It has been reported that the expression of transposable elements in germ cells was repressed by PRMT5 during embryonic stages^17^. However, the results of real-time PCR showed that the mRNA level of both *IAP-LTR* and *L1-ORF2* was not increased in *Prmt5*^*Δ/f*^; *Stra8-Cre* testes (Fig. S4)

### Symmetrical dimethylation on arginine-3 of histone H4 (H4R3me2s) was dramatically reduced in germ cells of *Prmt5* ^*Δ/f*^ *; Stra8-Cre* testes

**Figure 6:**
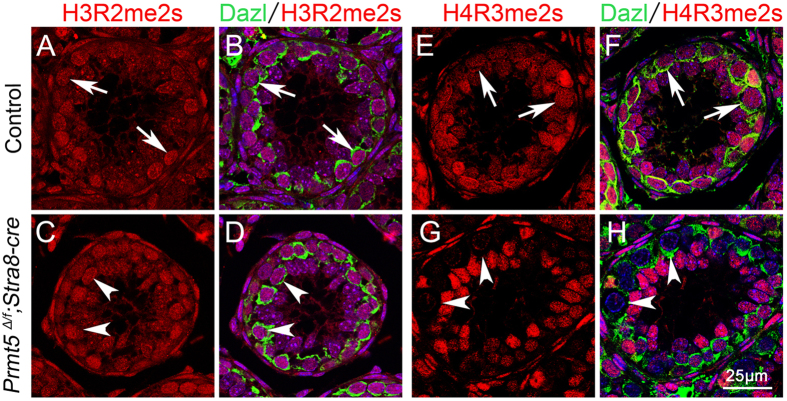
H4R3me2s was dramatically reduced in Prmt5-deficient germ cells. The histone methylation in control and *Prmt5*^*Δ/f*^*; Stra8-Cre* testes was examined by immunofluroresence at P10 . H3R2me2s was detected in germ cells of both control (**A**, **B**, white arrows) and *Prmt5*^*Δ/f*^*; Stra8-Cre* testes (**C**, **D**, white arrowheads). H4R3me2s was detected in germ cells of control testes (E, F, white arrows). By contrast, H4R3me2s signal was completely absent in the germ cells of *Prmt5*^*Δ/f*^*; Stra8-Cre* testes (**G**, **H**, white arrowheads).

As a protein arginine methyltransferase, PRMT5 is responsible for the symmetrical dimethylation of H4R3 in PGCs, and H4R3me2s is virtually absent in PGCs after *Prmt5* inactivation in mouse model^17^,^18^. H3R2me2s is another major form of histone methylation, which has been reported to be catalyzed by PRMT5^20^. To examine whether H4R3me2s and H3R2me2s are also catalyzed by PRMT5 in germ cells during spermatogenesis, immunofluorescence experiments were performed. As shown in Fig. 6, H3R2me2s was detected in germ cells of both control (A,B, white arrows) and *Prmt5*^*Δ/f*^*; Stra8-Cre* testes (C,D, white arrowheads) at P10. Robust signal of H4R3me2s was detected in germ cells of control testes (E,F, white arrows), whereas no signal was observed in the germ cells of *Prmt5*^*Δ/f*^*; Stra8-Cre* testes (G,H, white arrowheads) at this stage.

## Discussion

The functions of epigenetic modification in germ cell development have been reported previously. DNA methylation plays critical roles in retrotransposon silencing and germ cell development, deletion of *Dnmt3L* in mouse model up-regulates the transcription of *LINE* and *IAP* retrotransposons in spermatogonia and spermatocytes. The mutant mice display meiotic failure with widespread non-homologous chromosome synapsis and progressive loss of germ cells by the mid-pachytene stage^21^,^22^,^23^. The precise timing of establishment and removal of histone methylation marks is also critical for spermatogenesis. Double knockout of the H3K9 trimethyltransferase genes *Suv39h1* and *Suv39h2* causes defects in meiosis in male germ cells^24^. *Prmd9* (PR domain-containing 9) is an H3K4 trimethyltransferase that is specifically expressed in early meiotic germ cells both in the testis and ovary. Analysis of *Prdm9*-null mice indicates that it is involved in germ cell meiosis by regulating synapsis formation and recombination of homologous chromosomes during meiotic prophase^25^. Reduction of the histone methyltransferase MII2 activity results in a dramatic decrease of the number of spermatocytes by an apoptotic process^26^. Homozygous mutation of *Ehmt2* (euchromatic histone-lysine N-methyltransferase 2) in germ cells causes the arrest of meiosis at the early pachytene stage in both testis and ovary, indicating that H3K9 mono- and dimethylation is also essential for early meiotic progression^25^.

*Prmt5* encodes a protein arginine methyltransferase which catalyzes the symmetric dimethylarginine (SMDA) in both glycine and arginine-rich protein motifs. Previous studies have demonstrated that *Prmt5* is expressed in PGCs during embryonic stages. Specifically deletion of this gene in PGCs by crossing with *Blimp1-Cre* causes germ cell loss before sex determination. Kim *et al.* found that *Prmt5* functions in PGCs development by repressing transposable elements and that inactivation of *Prmt5* results in the up-regulation of *IAP* and *LINE1*^17^, whereas Li’s study revealed that *Prmt5* is required for germ cell survival by enabling the correct splicing of primary RNA transcripts that function in the DNA damage response pathway^18^.

In this study, we found that *Prmt5* was continually expressed in the germ cells of adult testis. Deletion of this gene after birth caused massive germ cell loss, and only a few germ cells were noted in adult testes. These results indicate that *Prmt5* is also required for postnatal germ cell survival in testis which is consistent with the functions during embryonic stages^17^,^18^. *Stra8-Cre* is reported to be activated in germ cells of testis at approximately 3 days after birth^19^, and we also found that both the mRNA and protein level of *Prmt5* were virtually absent in germ cells at P7. However, the development of germ cells was grossly normal in *Prmt5*^*Δ/f*^*; Stra8-Cre* testes at P10, *Stra8*-positive and *Scp3*-positive germ cells were observed in both control and *Prmt5*-deficient testes, indicating that the initiation of meiosis is not affected in *Prmt5*-deficient germ cells. The defect of germ cell development was first noted at P12 in *Prmt5*^*Δ/f*^*; Stra8-Cre* testes. In control mice, the number of germ cells was significantly increased at P12, and *Stra8*-positive and *Scp3*-positive germ cells were noted in the lumen of most seminiferous tubules. By contrast, most of the germ cells were still localized at the peripheral region of seminiferous tubules in *Prmt5*-deficient testes at this stage. A small number of *Stra8*-positive and *Scp3*-positive germ cells were detected in *Prmt5*-deficient testes. However, multiple foci of γH2AX were noted in *Prmt5*-deficient germ cells. In control germ cell, γH2AX was only detected in sex bodies. These results suggest that the DNA double strain breaks during meiosis are not properly repaired in *Prmt5-*deficient germ cells. Immunofluorescence results also showed that Prmt5 protein was mainly expressed in spermatocytes, not in germ cells at other developmental stages. Based on these results, we speculate that *Prmt5* is probably not involved in the development of germ cells before meiosis, but it is required for the process of meiosis. The death of *Prmt5*-deficient germ cells is probably due to the defects of meiosis. However, we could not exclude the possibility that the blockage of meiosis is a deleterious effect after *Prmt5* inactivation.

Previous studies found that both H4R3me2s and H3R2me2s are catalyzed by PRMT5^16^,^20^. In this study, we found that H4R3me2s was virtually absent in germ cells after *Prmt5* inactivation, whereas H3R2me2s was not changed in *Prmt5*-deficient germ cells, suggesting that PRMT5 is responsible for H4R3me2s in germ cell during later developmental stages. The functions of *Prmt5* in germ cell development are probably mediated by H4R3me2s which are consistent with other histone modifiers^24^,^25^,^26^. However, whether *Prmt5* is involved in germ cell development mediated by histone methylation or indirectly catalyzed the methylation of other proteins is unclear which need further investigation.

It has been reported that *Prmt5* is required for germ survival during embryonic stage by repressing the transcription of retrotransposon^17^. In this study, we found that the transcription level of *IAP* and *LINE1* was not increased in *Prmt5*-deficient germ cells, suggesting that *Prmt5* is required for germ cell survival at different developmental stages but the underlying mechanisms are different.

Taken together, in the present study, we found that *Prmt5* was also expressed in male germ cells after birth which also plays important roles in spermatogenesis. Deletion of this gene results in aberrant spermatogenesis and most of germ cells die in adult testes. The results suggest that *Prmt5* is probably required for germ cell meiosis and the death of *Prmt5*-deficient germ cells is likely due to the defects of meiosis. However, we could not exclude that the defect of meiosis is a deleterious effects after *Prmt5* inactivation.

## Material and methods

### Mice

All animal works were carried out in accordance with the protocols approved by the Institutional Animal Care and Use Committee at the Institute of Zoology, Chinese Academy of Sciences (CAS). All the mice were maintained in a C57BL/6;129/SvEv mixed background. *Prmt5*^*flox*^ mice strain was obtained from the European Conditional Mouse Mutagenesis Program (EUCOMM; *Prmt5*^tm2a(EUCOMM)Wtsi^)^27^, *Prmt5*^*Δ/+*^mice were obtained by crossing with *ZP3-Cre* mice. *Prmt5*^*Δ/f*^*; Stra8-Cre* mice were obtained by crossing *Prmt5*^*Δ/+*^*; Stra8-Cr*e males with *Prmt5*^*flox/flox*^ females. Genotyping was performed by PCR as described previously using DNA isolated from tail tips^28^,^29^.

### Tissue collection and histological analysis

Testes were dissected from *Prmt5*^*Δ/f*^*; Stra8-Cre* and control mice immediately after euthanasia, fixed in 4% paraformaldehyde for up to 24 hrs, stored in 70% ethanol, and embedded in paraffin. Five-micrometer-thick sections were cut and mounted on glass slides. After deparaffinization, sections were processed for immunohistochemistry and immunofluorescence analysis.

### Immunohistochemistry analysis

IHC analysis of tissues from at least three mice for each genotype was performed using a Vectastain ABC (avidin–biotin–peroxidase) kit (Vector Laboratories, Burlingame, CA) as recommended and using antibodies to MVH (1:500, Abcam, ab13840), PRMT5 (1:200, Millipore, 07-405). The IHC procedure was performed as described previously^28^. After staining, the sections were examined with a Nikon Microscope, and images were captured with Nikon DS-Ri1 CCD camera.

### Immunofluorescence analysis

After rehydration and antigen retrieval, the 5 μm sections were incubated with 5% donkey serum in 0.3% triton X-100 for 1 hr. Then the sections were incubated with the primary antibodies for 1.5 hrs, and the corresponding TRITC-conjugated donkey anti-rabbit IgG (1:150, Jackson) and FITC-conjugated donkey anti-mouse IgG (1:150, Jackson) for 1.5 hrs at room temperature. The following dilutions of primary antibodies were used: PRMT5 (1:200, Millipore, 07-405), DAZL (1:100, AbD Serotec, MCA2336), STRA8 (1:200, Abcam, ab49405), SCP3 (1:200, Abcam, ab15093), γH2AX (1:400, Millipore, 05-636), DMC1 (1:100, Santa Cruz, sc-22768 ), H3R2me2s (1:50, Millipore, ABE460), H4R3me2s (1:100, Abcam, ab5823). After three times wash in PBS, the nuclei was stained with DAPI. The sections were examined with confocal laser scanning microscope (Carl Zeiss Inc., Thornwood, NY).

### Nucleic acid isolation and quantitative reverse transcription PCR

The testes were dissected at P10, and stored at −80 °C after snap frozen with liquid nitrogen. Total RNA was extracted using a Qiagen RNeasy kit in accordance with the manufacturer’s instructions. Two micrograms of total RNA was used to synthesize first-strand cDNA. To quantify gene expression, real-time SYBR Green assay was performed with the isolated RNA. *Gapdh* was used as an endogenous control. All gene expression was quantified relative to *Gapdh* expression. The relative concentration of the candidate gene expression was calculated using the formula 2^−ΔΔCT^ as described in the SYBR Green user manual. Primers used for real-time PCR were listed in Table S1.

### TUNEL assay

TUNEL assays were conducted with the *In Situ* Cell Death Detection Kit, Fluorescein (Promega BioSciences, San Luis Obispo, CA, USA), as recommended. The images were captured by Nikon DS-Ri1 CCD camera.

### Statistical analysis

Experiments were repeated at least three times. Three to five control or *Prmt5*-deficient gonads at each time point were used for immunostaining. The quantitative results are presented as mean ± SEM. The data were evaluated for statistical differences using student T-test. P-value < 0.05 was considered as significant.

## Additional Information

**How to cite this article**: Wang, Y. *et al.* Prmt5 is required for germ cell survival during spermatogenesis in mice. *Sci. Rep.* **5**, 11031; doi: 10.1038/srep11031 (2015).

## Supplementary Material

Supplementary Information
