# Supplementary material for: Prmt5 is required for germ cell survival during spermatogenesis in mice
Source: Sci Rep. 2015 Jun 15;5:11031. doi: 10.1038/srep11031 (PMC4466585; doi:10.1038/srep11031)
Supplement: Supplementary Information [file srep11031-s1.pdf]

Prmt5 is required for germ cell survival during spermatogenesis in mice

Yanbo Wang[1,2], Tianxiang Zhu[4,1], Qiuling Li[3], Chunyi Liu[3], Feng Han[1,2], Min Chen[1,2], Lianjun Zhang[1,2], Xiuhong Cui[1], Yan Qin[1,2], Shilai Bao[3*], Fei Gao[1*]

[1]State Key Laboratory of Reproductive Biology, Institute of Zoology, Chinese Academy of Sciences, Beijing 100101, China

[2]University of Chinese Academy of Sciences, Beijing, China

[3]State Key Laboratory of Molecular and Developmental Biology, Institute of Genetics and Developmental Biology, Chinese Academy of Sciences, Beijing 100101, China

[4]School of Medicine, Zhejiang University, Hangzhou 310058, China

* Correspondence to: Fei Gao, gaof@ioz.ac.cn； or slbao@genetics.ac.cn

Figure S1

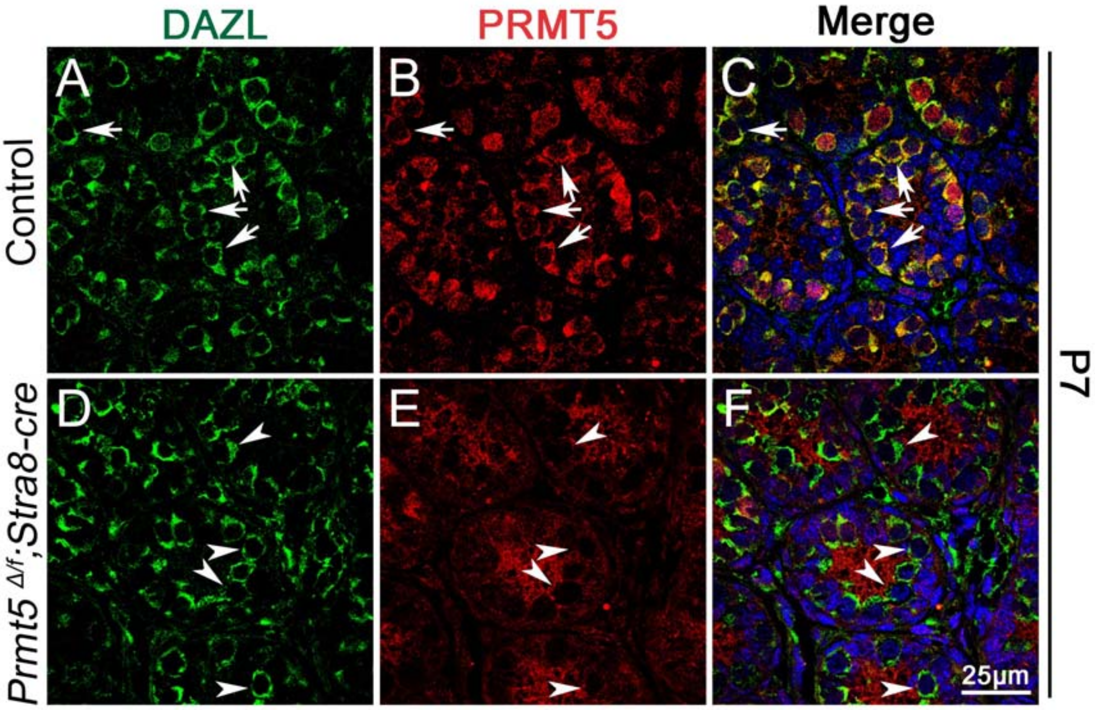

Figure S1. Prmt5 was inactivated as early as P7 in germ cells *of Prmt5$^{\Delta/f}$*; *Stra8-Cre* testes. The expression of Prmt5 (red) in control and *Prmt5$^{\Delta/f}$*; *Stra8-Cre* testes was examined by Immunofluorescence at P7. Germ cells were labeled with antibody against Dazl (green, white arrows). In control testes, Prmt5 was detected in cytoplasm of germ cells (B, C, white arrows), whereas no Prmt5 was detected in germ cells of *Prmt5$^{\Delta/f}$*; *Stra8-Cre* testes (E,F, white arrowheads).

Figure S2

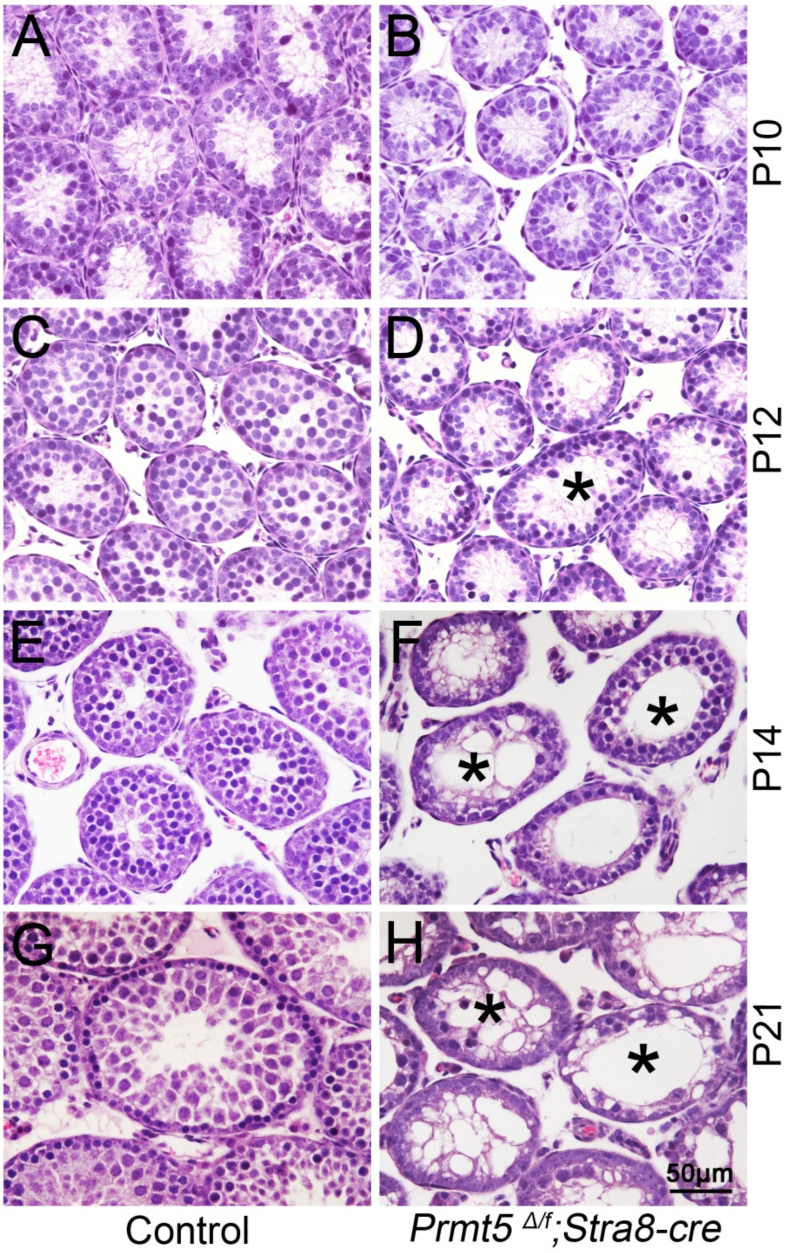

Figure S2. The defect of germ cell development was first observed in *Prmt5$^{Δ/f}$*; *Stra8-Cre* mice at P12. The results of H&E staining showed that the testes from *Prmt5$^{Δ/f}$*; *Stra8-Cre* mice (B) was grossly normal compared to control testes (A) at P10. Aberrant seminiferous tubules (asterisks) were first noted in *Prmt5$^{Δ/f}$*; *Stra8-Cre* testes (D, asterisks) at P12, and atrophic tubules (asterisks) were observed in *Prmt5*-deficient testes at P14 (F, asterisks) and P21 (H, asterisks).

Figure S3

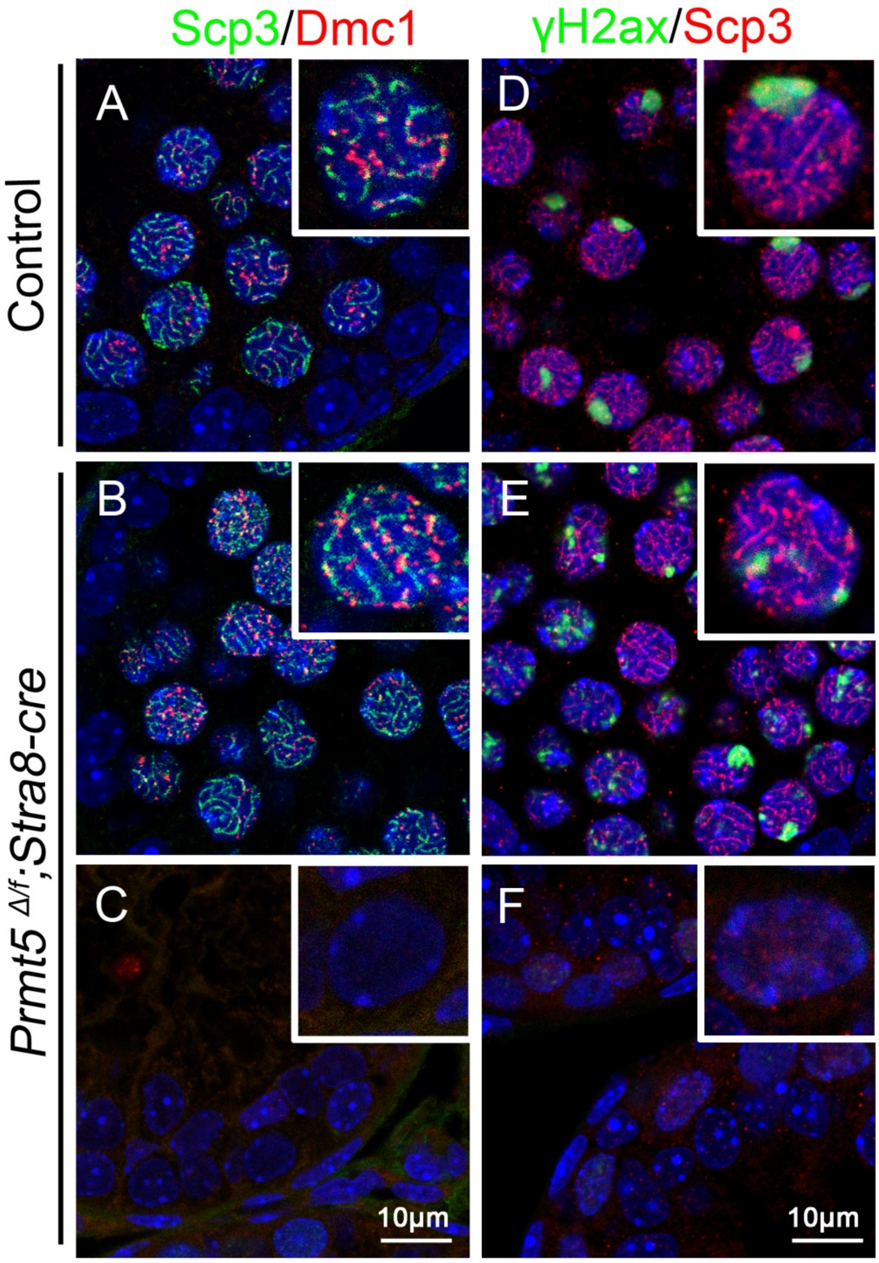

Figure S3. Immunofluorescence of Dmc1 and γH2AX. The expression of Dmc1 and γH2AX was examined by immunofluorescence. In control testes, Dmc1 protein (A, red) was detected in most of spermatocytes at P12, and co-localized with Scp3 (A, green). γH2AX (green) was mainly observed in sex body (D) in control testes. In *Prmt5*$^{\Delta/f}$*; Stra8-Cre* testes, Dmc1 protein (B, red) was only detected in a small number of germ cells, which was co-localized with Scp3 (B, green). γH2AX (green) was also detected in a small number of germ cells of *Prmt5*$^{\Delta/f}$*; Stra8-Cre* testes, whereas multiple foci were noted (E). Dmc1 (C) and γH2AX (F) protein was absent in most of germ cells from *Prmt5*$^{\Delta/f}$*; Stra8-Cre* testes.

Figure S4

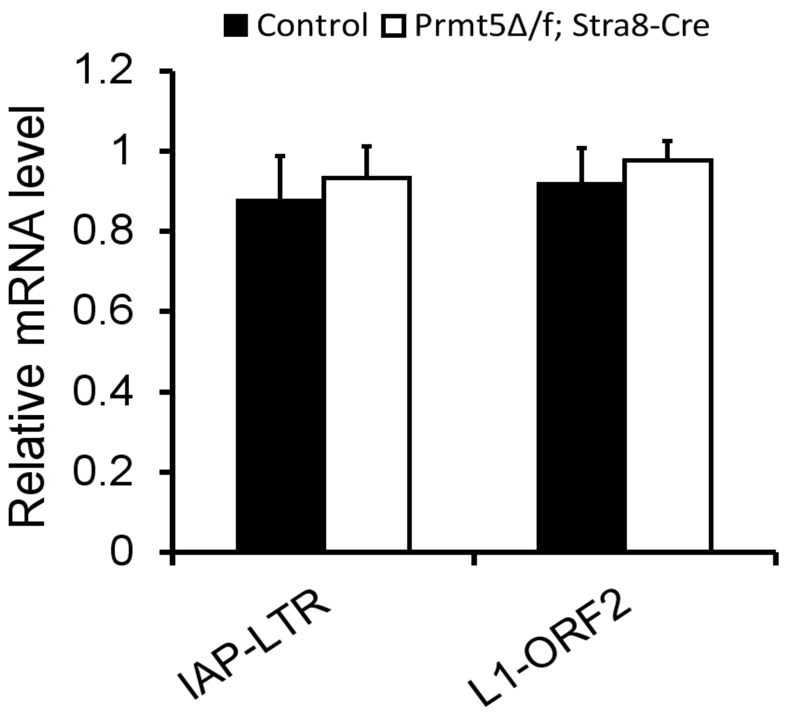

Figure S4. Loss of *Prmt5* did not result in up-regulation of transposable elements in germ cells. The expression of TEs was in testes at P10 was examined by real-time PCR. Compared to control testes, the mRNA levels of *IAP-LTR* and *L1-ORF2* in *Prmt5*$^{\Delta/f}$; *Stra8-Cre* testes were not increased.

Table S1

Gene	Forward primer sequence	Reverse primer sequence
Prmt5	TGGTGGCATAACTTTCGGACT	TCCAAGCCAGCGGTCAAT
Dazl	ATGTCTGCCACAACTTCTGAG	CTGATTTCGGTTTCATCCATCCT
Vasa	CTAGGAAGACCAAATAGTGAATCTGAC	TCCAGAACCTGTTACTACTTCTTCATT
Plzf	CCCAGTTCTCAAAGGAGGATG	TTCCCACACAGCAGACAGAAG
Stra8	CTGTTGCCGGACCTCATGG	TCACTTCATGTGCAGAGATGATG
Dmc1	CCCTCTGTGTGACAGCTCAAC	GGTCAGCAATGTCCCGAAG
Spo11	CGTGGCCTCTAGTTCTGAGGT	GCTCGATCTGTTGTCTATTGTGA
Rad51	GTCCACAGCCTATTTCACGGT	ACAGCCTCCACTGTATGGTAAC
Rec8	CTACCTAGCTTGCTTCTTCCCA	GCCTCTAAAAGGTGTCGAATCTG
Sycp3	AGAAATGTATACCAAAGCTTCTTTCAA	TTAGATAGTTTTTCTCCTTGTTCCTCA
Sycp1	AAGTTTGATTCTAAAACAACTCCTTCA	ACTCTTTTTAGTTGGTGTCTTCACTGT
Caspas3	GCTGACTTCCTGTATGCTTAC	ATTCCGTTGCCACCTTCC
L1-ORF2	CGGGAGACAGCACATACTAGCA	TCTCAAAGAACCAACTCCTCGT
IAP-LTR	ACATTCGCCGCCACAAGA	TAGTCGTAAATACCCTTGGCTCAT

Table S1. The primers used for real-time PCR analysis.
